# Assessment of a multi-cytokine profile by a novel biochip-based assay allows correlation of cytokine profiles with clinical outcomes in adult recipients of umbilical cord blood transplantation

**DOI:** 10.1038/s41409-019-0707-x

**Published:** 2019-10-02

**Authors:** Theodoros Karantanos, Haesook T. Kim, Natalia M. Tijaro-Ovalle, Lequn Li, Corey Cutler, Joseph H. Antin, Karen K. Ballen, Jerome Ritz, Ioannis Politikos, Vassiliki A. Boussiotis

**Affiliations:** 1 Hematology-Oncology, Beth Israel Deaconess Medical Center, Harvard Medical School, Boston, MA USA; 2 Medical Oncology, Johns Hopkins University, Sidney Kimmel Comprehensive Cancer Center, Baltimore, MD USA; 3 Dana-Farber Cancer Institute, Boston, MA USA; 4 University of Virginia Health Center, Charlottesville, VA USA; 5 Adult Bone Marrow Transplant Service, Department of Medicine, Memorial Sloan Kettering Cancer Center, New York, NY USA

**Keywords:** Translational research, Bone marrow transplantation

To the Editor,

Several studies have documented the association between cytokine levels after hematopoietic stem cell transplantation (HSCT) and infections, graft-versus-host disease (GVHD) and veno-occlusive disease, all of which represent main causes of nonrelapse mortality. Correlations between TNF-α or IL-1 levels and development of acute GVHD (aGVHD) are well described [1, 2]. Elevated IL-6 levels have also been associated with aGVHD and chronic GVHD (cGVHD), as well as early transplant-related complications [3]. IL-8 levels have been correlated with persistent neutropenia and graft failure, representing a predictive marker of bacteremia in neutropenic patients after HSCT [3]. Development of methods permitting the simultaneous measurement of multiple cytokines would improve our understanding of the kinetics and biological relevance of these correlations, and could lead to the development of biomarkers of high prognostic significance for post-transplant complications.

The simultaneous quantification of multiple cytokines in a single small-volume sample, presents a significant technical challenge because of the high detection threshold of the currently available methods that hampers these assessments in patients’ samples, particularly for the cytokines present at low concentrations [4]. We employed a novel method, which allows simultaneous measurement of multiple cytokines in 5 μl of plasma, and investigated retrospectively the prognostic value of a multi-cytokine profile in 27 adult recipients of double-unit umbilical cord blood transplantation (UCBT) following a reduced intensity conditioning regimen (Fludarabine, Melphalan, and ATG). The median patient age was 48 years and all patients had hematologic malignancies. IL-1β, IL-2, IL-4, IL-5, IL-6, IL-8, IL-10, IL-12, IL-17, IFN-γ, TNF-α, and GM-CSF were measured in samples obtained pretransplant and at 4, 8 weeks, 100 days, 6 and 12 months after UCBT, using the LUNARIS^TM^ Human 12-Plex Cytokine Biochip^384^ from AYOXXA Biosystems. Cytokine levels followed different posttransplant kinetics and were grouped into five patterns: (1) circulating IL-2 levels remained invariantly low; (2) IL-1β and IL-17 levels decreased over time; (3) TNF-α levels increased at 1 month after UCBT and remained stable; (4) IL-4, IL-5, IL-6, IL-8, IL-10, IL-12, and GM-CSF levels peaked at 1 month after UCBT and subsequently declined to pretransplant levels; (5) IFN-γ levels peaked at 2 months after UCBT and subsequently declined. Spearman correlation matrix showed that most of the cytokines were positively correlated with each other during the first 12 months after UCBT with the exception of IL-8, which positively correlated only with IL-6. Validation was performed by assessing half of the cytokines by the standard ELISA.

**Fig. 1:**
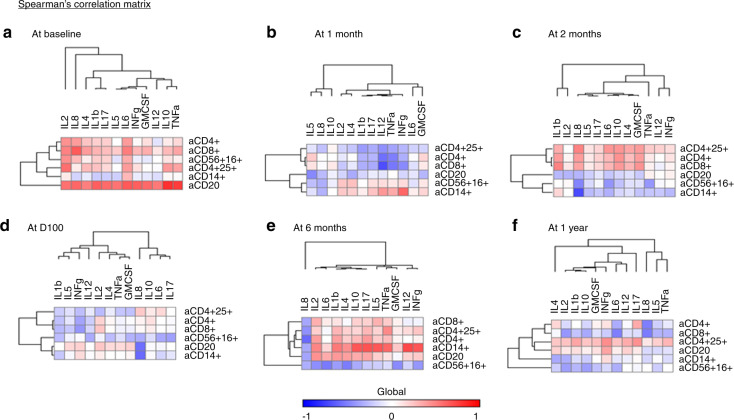
Spearman correlation between cytokines and counts of different white blood subtypes. Distinct correlations between individual cytokines and absolute counts of white blood cell subsets were observed before and at various time points after UCBT (see text for detailed description). All *p*-values in the Spearman matrices were corrected for multiple comparisons

We assessed the correlation between cytokine levels and reconstitution of white cell subtype counts after transplantation (Fig. 1). At baseline all cytokines were positively correlated with B cells (CD20^+^). IL-2 was also positively correlated with T regulatory and CD8^+^ cell counts. CD8^+^ cell counts were correlated with IL-6 and IL-8 levels. At 1 month after UCBT, IL-2 levels were negatively correlated with CD8^+^ cells, and TNF-α levels were negatively correlated with CD4^+^ and CD8^+^ cell counts. At 2 months, IL-8 levels were negatively correlated with CD56^+^CD16^+^ and CD14^+^ cell counts and positively correlated with T regulatory cells. At 100 days and 6 months following UCBT, IL-8 levels were negatively correlated with CD20^+^ and CD4^+^ cell counts, respectively. In contrast, at 6 months, CD14^+^ monocyte counts were positively correlated with IL-10, IL-12, IL-17, IFN-γ, and TNF-α, likely representing the activation of reconstituted monocytes producing inflammatory cytokines. No statistically significant correlations between cytokine levels and white cell subtypes were observed at 12 months after UCBT. IL-6 levels were higher in the cGVHD cohort at 6 and 12 months after UCBT (*p* = 0.046 and *p* = 0.023, respectively). IL-8 levels trended similarly, but the differences did not reach statistical significance.

Our report provides a new paradigm that biologically meaningful levels of multiple cytokines can be measured simultaneously in a very small volume of patients’ plasma. The results obtained in our study using this novel technique are consistent with previous observations generated using standard approaches of cytokine assessment. We found that inflammatory cytokines such as IFN-γ, IL-6, and TNF-α are increased at 1 to 2 months after UCBT, followed by a subsequent decline, with the exception of IFN-γ, which remained relatively stable. These findings are in agreement with previous studies [3, 5]. During the same posttransplant period, the levels of inhibitory cytokines, such as IL-10, have been positively correlated with the levels of inflammatory cytokines, probably representing a compensatory response [6]. Indeed, we observed that cytokines implicated in Th1 inhibition, such as IL-4, IL-5, and IL-10, were increased at 1 month after UCBT followed by a subsequent decline. We found that IL-6 levels during the posttransplant period positively correlated with the development of cGVHD. Elevated levels of IL-6 have been previously associated with aGVHD and cGVHD [3]. In our UCBT cohort, the incidence of aGVHD was very low, thereby explaining why correlation of IL-6 was only detected with cGVHD. Notably, our study showed that the levels of IL-1β were very low during the entire time and together with the levels IL-17 declined after UCBT as compared with pretransplant levels. This kinetics profile differs from that observed in recipients of adult HSCT, who display increase of IL-1β and IL-17 levels after transplantation and correlation of these cytokines with aGVHD [2]. This difference is in agreement with the low rate and severity of aGVHD in our cohort.

Although our sample size is small, our observations provide evidence that the novel method that we employed generates biologically and clinically meaningful results. This approach can be useful for identification of biomarkers with diagnostic and prognostic significance, not only in HSCT, but also in other conditions in which cytokine production is the mediator or the side effect of therapy. For example, our approach may allow simultaneous assessment of multiple pro-inflammatory cytokines in patients who receive immunotherapies with monoclonal antibodies, bispecific antibody blinatumomab engaging CD19 and CD3, or chimeric antigen receptor T (CAR-T) cells. The most prevalent adverse effect of such immune-based therapies is the syndrome of immune activation, known as cytokine release syndrome (CRS) [7]. The hallmark of CRS is the elevation of inflammatory cytokines including IFN-γ, GM-CSF, IL-10, IL-6, and IL-1 [7–11]. The technical difficulties of monitoring serum cytokines in real time have precluded the application of this approach to identify evolving CRS. Currently, the use of IL-6 receptor blockade with tocilizumab is the mainstay of CRS treatment [7, 8, 12]. However, it is unclear whether blockade of IL-6 receptors might affect the function of CAR-T cells and their anti-tumor efficacy. Furthermore, IL-1 rather than IL-6 might be the key inflammatory cytokine responsible for CRS [10, 11]. Simultaneous measurement of all cytokines tentatively involved in the pathophysiology of CRS will allow determination of their precise and selective kinetics, their association with CRS and other complications of immune-based therapies, and their correlation with therapeutic outcomes. Thus, the novel method described in this report might be a highly appropriate tool to monitor the outcomes of HSCT as well as the efficacy and complications of modern immunotherapies.
